# Vertical Optical Scanning with Panoramic Vision for Tree Trunk Reconstruction

**DOI:** 10.3390/s17122791

**Published:** 2017-12-02

**Authors:** Adilson Berveglieri, Antonio M. G. Tommaselli, Xinlian Liang, Eija Honkavaara

**Affiliations:** 1 Department of Cartography, São Paulo State University UNESP, 305, Presidente Prudente 19060-900, Brazil; tomaseli@fct.unesp.br; 2 Department of Remote Sensing and Photogrammetry, Finnish Geodetic Institute FGI, National Land Survey of Finland, 00521 Helsinki, Finland; xinlian.liang@nls.fi (X.L.); eija.honkavaara@nls.fi (E.H.); 3 Centre of Excellence in Laser Scanning Research, Academy of Finland, 02430 Helsinki, Finland

**Keywords:** dense image matching, fisheye camera, photogrammetry, 3D point cloud, structure from motion, diameter at breast height, DBH, tree trunk

## Abstract

This paper presents a practical application of a technique that uses a vertical optical flow with a fisheye camera to generate dense point clouds from a single planimetric station. Accurate data can be extracted to enable the measurement of tree trunks or branches. The images that are collected with this technique can be oriented in photogrammetric software (using fisheye models) and used to generate dense point clouds, provided that some constraints on the camera positions are adopted. A set of images was captured in a forest plot in the experiments. Weighted geometric constraints were imposed in the photogrammetric software to calculate the image orientation, perform dense image matching, and accurately generate a 3D point cloud. The tree trunks in the scenes were reconstructed and mapped in a local reference system. The accuracy assessment was based on differences between measured and estimated trunk diameters at different heights. Trunk sections from an image-based point cloud were also compared to the corresponding sections that were extracted from a dense terrestrial laser scanning (TLS) point cloud. Cylindrical fitting of the trunk sections allowed the assessment of the accuracies of the trunk geometric shapes in both clouds. The average difference between the cylinders that were fitted to the photogrammetric cloud and those to the TLS cloud was less than 1 cm, which indicates the potential of the proposed technique. The point densities that were obtained with vertical optical scanning were 1/3 less than those that were obtained with TLS. However, the point density can be improved by using higher resolution cameras.

## 1. Introduction

Tree attributes can be obtained using different terrestrial techniques for 3D data acquisition. Laser scanning (LS), which is also referred to as Light Detection and Ranging (Lidar), has been widely used for commercial purposes in forest inventories. It can directly acquire dense and accurate 3D point clouds to provide a detailed and accurate description of the objects of interest.

LS systems, such as terrestrial laser scanning (TLS) and personal laser scanning (PLS), have been used to collect forest attributes. TLS produces high point densities by providing a large set of information on forest structural parameters. Studies have used TLS for forest data collection, including the estimation of tree height, plot- and tree-level volumes, forest canopy, trunk density, and diameter at breast height (DBH), as noted by Liang et al. [1]. Such data are important for monitoring of tree growth, timber volume estimation, biomass estimation, and forest inventory, for example. Furthermore, the DBH is an essential variable because it can be correlated with other measures that are more complicated to obtain such as biomass and wood volume.

Liang et al. [2,3] developed a PLS system for collecting tree attributes. The system is carried by a human operator who walks through the forest to collect PLS measurements. The system offers high mobility within the environment and rapid data collection. However, the positioning and orientation in dense forests are problematic due to the loss of satellite signals.

Image-based techniques can also be used to accurately collect tree attributes. Although stereoscopy has a long history of study and application, the automated 3D reconstruction of point clouds from stereo or multiple images has been limited due to its high computational load. The rapid evolution of hardware and improvements in algorithms (e.g., structure from motion (SfM) [4] and semiglobal matching [5]) have allowed the dense matching of large sets of images to be automated. This process enabled the use of multiple images as a source for generating 3D point clouds.

The use of terrestrial images for mapping forest plots and obtaining tree attributes has been studied with different approaches, such as using a film camera [6], a two- or multi-camera system [7,8], an uncalibrated hand-held consumer camera [2], stereoscopic hemispherical cameras [9,10], and a nadiral fisheye camera with a telescopic pole [11].

Liang et al. [12] compared methods of collecting forest data using TLS, PLS and photogrammetric techniques. The authors explored the possibility of using consumer cameras for data acquisition in forest plots and concluded that tree attribute could be estimated at a low cost and by non-professionals, such as individual forest owners, but the estimates were less accurate than those obtained with LS techniques.

Forest studies using fisheye cameras are not a novelty. Early research by Evans and Coombe [13] used fisheye cameras to investigate the sunlight penetration through forest-based environments in images. An important issue that complicates the use of fisheye lenses is the non-uniform ground sample distance (GSD). However, fisheye lenses provide the wide field of view that makes it possible to cover an entire scene with few images.

Currently, different mathematical models can be found for fisheye camera calibration and orientation [14,15,16], which enable the automation of fisheye image processing. The incorporation of fisheye geometric models into commercial software (e.g., Pix4D, ContextCapture, and PhotoScan) shows the potential of this type of geometry for both panoramic and metric applications. The level of automation of information extraction and processing with fisheye cameras is similar to that of projects that are based on central perspective cameras.

In this paper, the objective is to show that a previously developed vertical optical scanning technique with fisheye images can be used for dense reconstruction of tree trunks using commercially available software and off-the-shelf components. Vertical optical scanning was originally developed by Tommaselli and Berveglieri [17] and applied to accurately measure the diameters of tree trunks by Berveglieri et al. [11], including automatic trunk location. However, the reconstruction was sparse, so only trunk location and DBH measurement were possible. In previous papers, scientific non-commercial software programs (developed in-house) were used for the technical implementation. We expanded the potential of the technique by using dense matching algorithms, which have been previously implemented in commercial and open-source software.

The low cost of the required devices, the feasibility of technical implementation, and mainly the high density of the point cloud and its resulting accuracy are benefits that encourage the use of this technique for highly efficient remote sensing. Problems with occlusion are still a limitation but are not restricted to this technique, since other methods of data collection with optical cameras or even LS share the same problems. LS, however, has the advantage of pulse penetration in vegetation.

The aim of this work is to show that the 3D image-based point cloud has similar accuracy to the TLS cloud and a point density that is sufficient for extracting accurate measurements and details of the scene objects, as will be presented in the following sections.

## 2. Study Area and Data Acquisition

This section describes the area that was selected for collecting the images using the proposed technique and the methods that were applied for 3D reconstruction in a forest environment.

### 2.1. Test Area and Data Acquisition

A test area, which is located in Masala, Finland (60.15° N, 24.53° E), was selected for the experiments. This area is a forest plot that is composed of Scots pines (*Pinus sylvestris* L.), birches (*Betula* sp. L.), saplings, and shrubs. The tree density is 278 trunks/ha for a DBH > 10 cm, which is measured at approximately 1.30 m above the ground.

**Figure 1:**
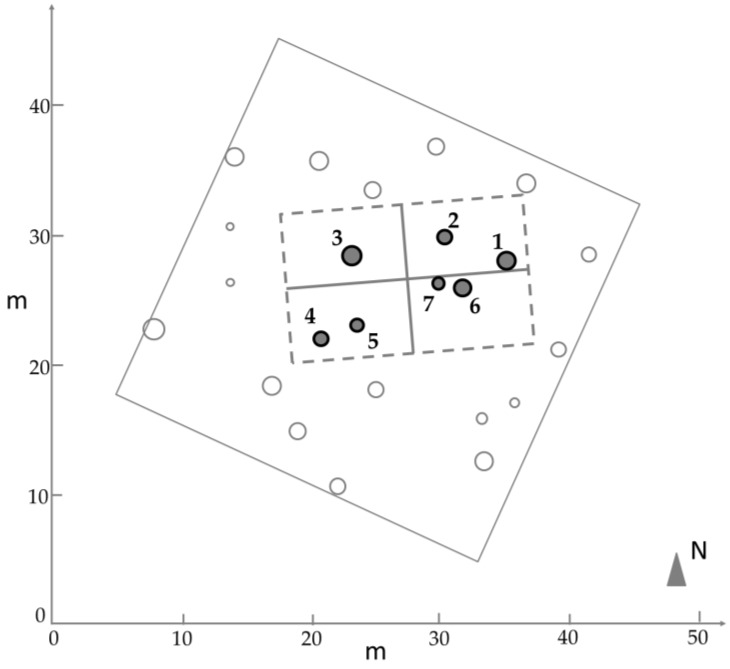
Forest plot that was used for the experiments. The dashed rectangle indicates the group of trees that were selected for 3D reconstruction.

Locations of the trees were measured inside the plot using a total station Trimble 5602 DR 200. A prism was positioned near the DBH height of each tree. Then, the distance and the vertical angle between the total station and the prism were measured to determine the tree position. Tree circumferences were measured using a measuring tape at the DBH. Optical images and TLS data were collected in approximately the same position inside the plot for experiments and comparison of the results. Figure 1 depicts the area that was used for the experiments.

#### 2.1.1. Image Data

**Figure 2:**
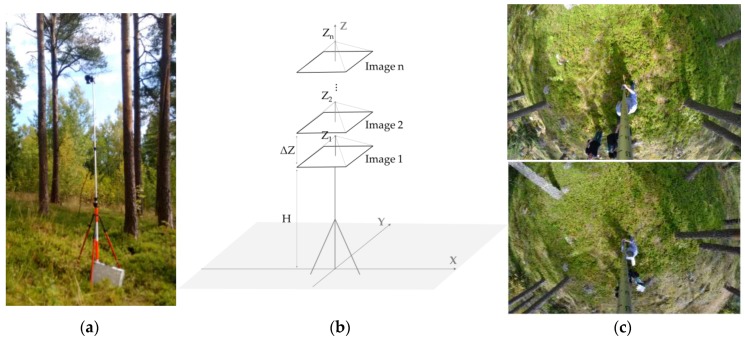
(**a**) Fisheye camera positioned in the nadir viewing position to collect images; (**b**) Camera displacement (ΔZ) for acquisition of a vertical image sequence (*n*); (**c**) Fisheye images in the nadir viewing position (first and last images in the sequence).

A fisheye camera (technical features are listed in Table 1, Nikon Inc., Melville, NY, USA) that was mounted in the nadir viewing position on a telescopic pole (i.e., the direction pointing directly down) was used to collect vertical images, as shown in Figure 2a. This positioning technique enables a large field of view over the scene with high overlap among images; therefore, fewer images must be taken compared to the requirements of other techniques. In the forest plot, the camera was focused to infinity to avoid problems caused by autofocus. The aperture size and ISO (International Organization for Standardisation; in this case, referring to sensor sensitivity) settings were adjusted according to the light conditions. Twenty-four vertical images were taken by varying the camera height (Z) from 3.40 m up to 4.60 m, which generated images with increasing GSDs. A remote control was used to trigger the camera.

As depicted in Figure 2b, the acquisition technique is implemented by displacing the camera vertically in small increments, thereby creating a vertical optical flow in which an image sequence is captured. First, the acquisition system is positioned in a suitable place in the forest plot and the camera is raised to an initial height (e.g., H = 3 m). Next, images are taken sequentially, starting with the first image and maintaining the same XY position but incrementing the camera height by ΔZ. Figure 2c shows two examples of fisheye images that were acquired in the nadir viewing position inside the test plot (the first and the last camera positions in the image sequence). Originally, Berveglieri et al. [11] used only three images (separated by approximately 50 cm in height) to obtain photogrammetric measurements. In this case, an image sequence with small displacements in height (as an optical flow) is used for 3D reconstruction.

To make the technique more practical by decreasing the time of image acquisition and height measurement, a telescopic pole with predefined markings can be used to move the camera vertically. From an initial height, the camera can be lifted to the fixed marks. Thus, the heights will always be known, as well as their increments, which are essential for setting the scale in the image block adjustment. Vertical optical scanning can be performed continuously until the desired height is reached.

#### 2.1.2. TLS Data Acquisition

TLS data were collected using a terrestrial laser scanner (Faro Photon3D X 330, Lake Mary, FL, USA), which has a ranging accuracy of 2 mm for a distance of up to 25 m. Table 2 summarizes the main technical details of the instrument.

The scanner was positioned inside the plot near the position that was used to collect the fisheye images. The TLS data were processed by the robust modelling method [1] to extract tree attributes. To eliminate influences as branch points, the points were weighted in the modelling process using the Tukey estimator.

In this case, reference targets for coregistration were not needed because only one single TLS station was used to collect the data. However, if the purpose is to produce a point cloud from multiple stations, reference targets can be arranged in the forest plot to be used for both image capture and TLS data acquisition.

## 3. Methods

An image-based point cloud was generated with the set of fisheye images. Then, tree parameters were estimated from this point cloud.

Various mathematical models for camera calibration and orientation are available for fisheye lenses. Accuracy evaluations of several fisheye models can be found in [15,16]. In this study, we used the Agisoft PhotoScan commercial software (Agisoft LLC, St. Petersburg, Russia) and adopted a fully automated workflow for point cloud generation, from camera calibration to 3D reconstruction.

PhotoScan uses the equidistant projection with the generic form, which is presented in (1), to determine the image coordinates (*x*, *y*):

(1) $$\begin{array}{l} x=\frac{f}{\sqrt{{\left(\frac{X}{Y}\right)}^{2}+1}}\arctan\left(\frac{\sqrt{X^{2}+Y^{2}}}{Z}\right)+c_{x}+\Delta x\\ y=\frac{f}{\sqrt{{\left(\frac{X}{Y}\right)}^{2}+1}}\arctan\left(\frac{\sqrt{X^{2}+Y^{2}}}{Z}\right)+c_{y}+\Delta y \end{array}$$

where *f* is the camera focal length, (*X*, *Y*, *Z*) are 3D object point coordinates, *c_x_* and *c_y_* are principal point offsets, and Δ*x* and Δ*y* represent additional parameters for compensating for the systematic errors in the image coordinates. These additional parameters include radial symmetric and decentring distortions [18] and parameters for modelling affinity and shear [19].

Then, the set of fisheye images was input into the photogrammetric project to enable point cloud generation using the automated image matching procedure of PhotoScan software. The procedure was performed to estimate the exterior orientation parameters (EOPs) and the interior orientation parameters (IOPs) using bundle block adjustment with the SfM algorithm.

For conventional cameras, PhotoScan software extracts initial data on focal length and pixel size from the header file. However, since this fisheye lens does not have this interface, the initial data had to be manually input into the software.

In this step, the camera heights that are recorded during image acquisition must also be inserted as weighted constraints for the *Z* coordinate. The *XY* coordinates can be assumed as (0, 0) in a local reference system and a constraint with a standard deviation of 5 cm can be adopted to estimate the planimetric camera positions. This weighting strategy is necessary because of minor planimetric displacements with respect to the *Z*-axis when raising the camera. The attitude angles can be determined based on the bundle adjustment as unknowns.

Since weighted constraints are imposed to enable a relative image orientation, the proposed technique does not require ground control points, which is an advantage when performing the field survey. Thus, the point cloud is generated in a local reference system by an image matching procedure. In the image alignment process, the full-resolution images were used and automated camera calibration was performed, along with image matching. The point cloud was generated using a high-point-density setting.

**Figure 3:**
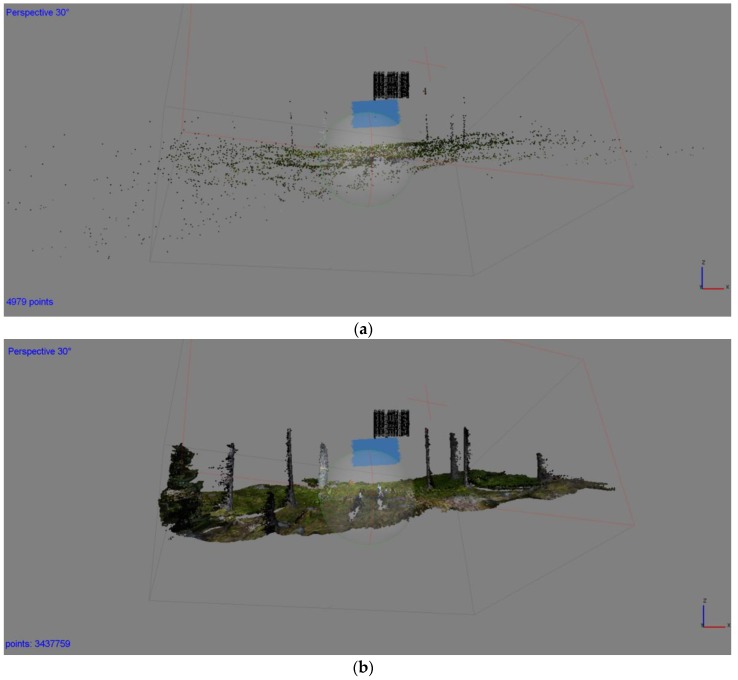
(**a**) Camera poses and tie points after bundle adjustment; (**b**) Dense point cloud.

Figure 3a displays the generated tie points (4979 points) and the camera poses after bundle adjustment. A dense point cloud with 3,437,759 points is presented in Figure 3b. The average camera location errors, which are provided by the PhotoScan report, are as follows: *X* error of 1.12 cm, *Y* error of 0.47 cm and *Z* error of 1.78 cm relative to the initial camera positions. Thus, the reconstruction quality was high, with a high-quality point cloud and precisely reconstructed trunks and ground cover.

Then, various data can be extracted from the generated point cloud, such as DBH sizes, geometric shapes of trunks, distances between trees, spatial distribution, and even a plot mapping. From this point cloud, circles or cylinders can automatically be fitted to extract trunk sections or DBH estimates, for example. Moreover, the data set is a realistic plot model.

## 4. Results

Several tests were conducted to assess the accuracy of the 3D reconstruction, since the quality of the image-based point cloud is influenced by the matching algorithm.

The circumferences of seven tree trunks were directly measured at the DBH with a measuring tape to obtain the reference values. The accuracy assessment was based on the discrepancy between the measured and estimated diameters. A vertical range of approximately 10 cm was extracted for each trunk in the 3D point cloud around the DBH height. As performed by Berveglieri et al. [11], from the *XY* planimetric coordinates, a circle was fitted to each trunk to estimate its centre and radius, from which the diameter can be obtained. This was done based on the assumption that usually tree trunks have a cylindrical shape, except for the base and top. Thus, a regular cylinder can be used as a geometric model for tree trunk.

**Figure 4:**
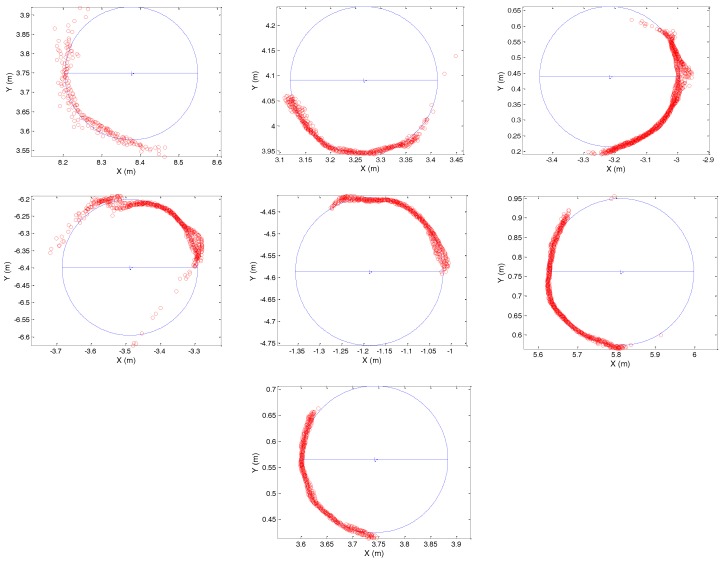
Circles fitted to the seven trunks to estimate the diameter at breast height (DBH) and position in the forest plot.

Figure 4 shows the seven circles that were fitted to the curvature of each trunk and the position of each trunk in the forest plot, considering that the position of the camera station was adopted as the origin of the reference system.

Table 3 presents the a posteriori sigma results of each circle fitting, which varied from 0.4 cm to 1.1 cm, and the errors in estimating the DBHs using the vertical optical scanning technique. The errors varied from 0.22 cm to 2.54 cm. The RMSE was calculated as 1.47 cm, which represents the overall accuracy of the image-based technique for this study case.

**Figure 5:**
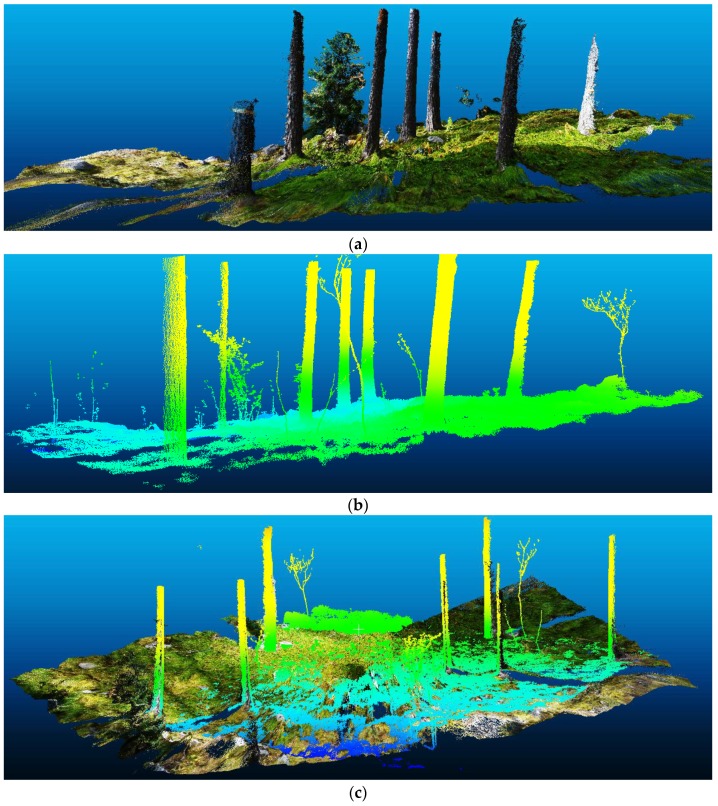
(**a**) Image-based point cloud; (**b**) TLS-based point cloud; (**c**) Overlap of the two point clouds.

The image-based point cloud in Figure 5a was compared with the TLS-based point cloud, which is shown in Figure 5b, to assess the differences in 3D trunk reconstruction. Figure 5c displays the overlap of the two point clouds to show that they are similar, except for a few bushes that did not exist when the images were collected.

**Figure 6:**
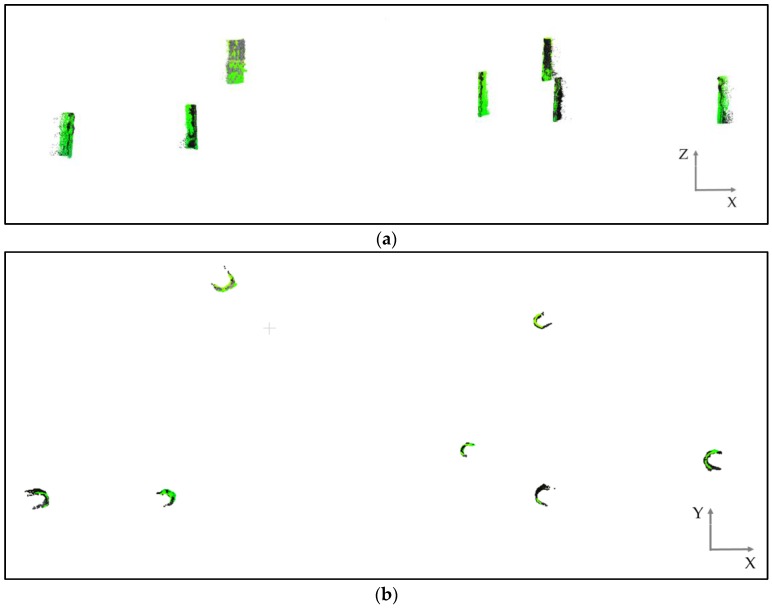
(**a**) Trunk sections extracted from the two point clouds; (**b**) Circumferences of the seven trunks in both point clouds.

To accurately assess the similarity between the point clouds, vertical sections of approximately 1 m were extracted from the trunks and cylinders were fitted to these segments, since the trunks have a cylindrical shape. Figure 6a presents cylindrical sections that were fitted to overlapping clouds to have the same cut length, and Figure 6b is a nadir view that shows the trunk curvatures.

The strategy for comparison was based on fitting a cylinder to each trunk automatically. Then, the difference between the estimated radii could be analysed. The algorithm that was used for point cloud shape detection was based on a high-performance random sample consensus (RANSAC), which was developed by Schnabel et al. [20]. The technique was applied to each point cloud separately.

Table 4 presents the data for the comparison of the trunk sections. As expected, the point density of each trunk section was larger in the TLS point cloud than in the image-based point cloud, except for trunk 6. On average, the point density with images was approximately 1/3 less than that with TLS. Regarding the differences between radii, only trunk 6 presented a value above 1 cm, which was due to the low density that was obtained by the TLS in this particular case. The mean of the differences was less than 1 cm, with an overall difference of approximately 0.63 cm between the diameters that were extracted from both point clouds.

The relative error in the positioning of the trees in the forest plot was assessed by comparing the tree axis that was extracted from the two point clouds. In this case, the trunk locations were defined based on the centres that were estimated by the circle fitting. A rigid-body geometric transformation was computed by the least-squares method based on the coordinates of the seven trees in both reference systems (optical scanning and TLS). Thus, an analysis could be performed based on the residuals to evaluate differences in the tree locations.

**Figure 7:**
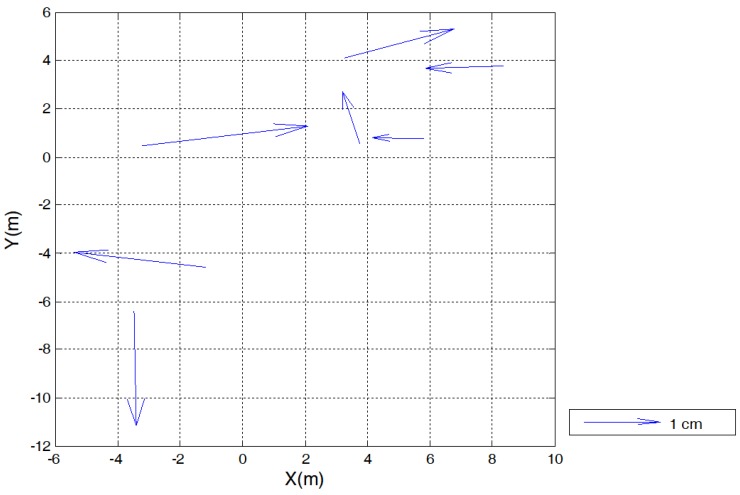
Needle map of the residuals resulting from the rigid-body transformation using the least-squares method.

Figure 7 depicts the needle map of the residuals in *XY* for each trunk, which shows random behaviour. The standard deviation of the residuals, which represents the relative error between the optical and TLS techniques for tree positioning, was calculated as 1 cm in *X* and 0.54 cm in *Y*. It is not possible to state which of these techniques is more accurate because there is no accurate ground reference. However, it can be concluded that vertical optical scanning provides a positioning precision that is comparable to that of TLS for the study case.

A test was also performed to assess the trunk reconstruction by the PhotoScan software when the height constraints that were measured in the field were not imposed on the *Z* coordinate. The purpose was to determine whether the SfM algorithm could accurately estimate the camera EOPs. Visually, the generated point cloud was similar to that obtained when constraints were imposed on the initial camera position parameters. However, the results were inaccurate due to scale distortions, with a total error of 9.6 m for the camera location.

## 5. Discussion

Image-based point clouds that are obtained by vertical optical scanning with a fisheye lens can be used for tree attribute extraction and mapping of forest plots. This study investigated the feasibility of acquiring fisheye image sequences with a precalibrated camera to obtain forest data and compared the quality of the image-based point cloud with a cloud that was obtained with TLS technique.

TLS- and image-based point clouds are acquired using different measurement principles. TLS has been the dominant technique for collecting 3D point clouds, but the advances in dense matching algorithms have increased the potential of image-based reconstruction techniques compared to LS techniques.

The optical system that is employed to capture fisheye images in a forest plot is of low cost, low weight, and small size and it is easy to handle for non-expert users. In contrast, TLS devices are expensive, heavy, and require a professional operator. Occlusion is also a limiting problem for both the optical technique and the TLS technique, although TLS pulses can penetrate much deeper into the vegetation. Although the operating range of the optical technique is smaller than that of a TLS system, measurements with better than 2 cm accuracy can be obtained for a radial distance of up to 10 m. However, this distance is only achievable in non-dense plots where trees are sparse.

The results that are reported in this paper show that the point cloud that is derived from the fisheye images has the potential to deliver accurate tree attributes. The results demonstrate that DBH and tree location in a forest plot can be estimated from an image-based point cloud using the proposed technique with accuracy that is acceptable for field inventories, for example.

In general, the accuracy of DBH estimation using TLS data is expected to be better than that using optical data. However, the accuracy that resulted from the image-based point cloud was similar to that obtained with a TLS point cloud using a single scan. The trunks that were reconstructed with image matching were visually realistic, and comparing them to the real measures, the overall accuracy was 1.47 cm in DBH estimates with circle fitting. A forest map can be generated by plotting the trunk centre coordinates that are obtained from the circle fittings, together with the estimated radii, and these coordinates can be transformed to a global coordinate system by geotagging the camera station and measuring the camera azimuth. The comparison of the tree positioning between the optical scanning and TLS techniques indicated an average difference of 1 cm in a local reference system.

The point density and geometric accuracy can vary in an image-based point cloud. Inaccurate camera calibration and image matching problems can produce sparse and noisy points and affect the quality of the generated point cloud. A comparison between the image-based and TLS point clouds was also performed using cylinder fitting based on RANSAC in trunk sections. Cylinders were fitted to similar sections between the point clouds to assess the 3D reconstruction based on the shape. The results of the comparison between the radii of the cylinders (image-based versus TLS) indicated a difference of less than 1 cm in six trunks and a value of 1.51 cm in one trunk. This represented a similarity level of 0.63 cm, on average.

The number of points in each trunk section was compared. Even with only 1/3 as many points as the TLS point cloud, the image-based point cloud produced accurate estimates. This was possible because well-distributed points over the trunks were generated, which allowed a detailed reconstruction of the trunk geometric shapes.

Inserting an accurate scale factor (known vertical displacement) is another important aspect of the 3D reconstruction by the dense image matching algorithm. To achieve that, the approximate camera position with weighted constraints must be provided for the *Z* coordinates (or scanning direction). The two tests that were performed with and without constraints on the camera position in the bundle adjustment showed the need for accurate *Z* values, which adjust the scale to produce an accurate point cloud. As the fisheye technique utilised vertical optical scanning, the height measures that were marked on the telescopic pole were used as values for *Z*.

In this experiment, the camera was lifted manually. However, an automated mechanism could be developed to achieve higher efficiency and even higher accuracy. Another advantage of this system is the small field area that is required to install and operate it, which is important when walking is difficult within a plot. Liang et al. [2] discussed the difficulty of collecting data within forest plots using standard surveying techniques. Thus, the proposed technique with vertical optical scanning can also facilitate data acquisition in forest plots. The heights of points that were reconstructed by the proposed technique were limited to the camera height. Depending on the height that is achievable by the camera, and other tree trunk parameters can be estimated from this point cloud, e.g., straightness or curvature of trunk. A full 360° field of view can be achieved attaching a second camera that points upward. Assessing this option was left for future work.

## 6. Conclusions

This paper presented a technique named vertical optical scanning and detailed experimental results of a field test, which demonstrate its applicability in a forest plot for collecting tree attributes and mapping. We assessed the feasibility of this technique with a fisheye camera and image processing with photogrammetric software using the Agisoft PhotoScan commercial software package, which uses the SfM algorithm for 3D reconstruction. Our aim was to show that the proposed technique can be used in a simple way with available software for high-quality dense reconstruction. The high number of overlapping images and the constraints that are imposed on the camera positions make it possible to generate a dense, visually realistic and accurate point cloud from which tree attributes can be extracted. The generated dense point cloud (obtained with dense matching) reproduces the characteristics of local objects in detail. Therefore, the extended shape of a trunk can be obtained, as well as structures of branches and even details on the ground.

The vertical optical scanning technique does not depend on control points or signalised targets. Its accuracy relies on the constraint set in the scanning direction (*Z*-axis in this study), which can be marked on the telescopic pole. The vertical sequence of overlapping images provides a robust geometry for the estimation of the camera poses and dense image matching. Because of the accurate results, the quality of the data that are generated with the fisheye technique is comparable to the quality of the data that are produced by a single TLS system. The tree location is produced in a local reference system, which typically occurs in terrestrial surveys of forests due to the low-quality satellite signals inside forests, which can be easily interrupted.

The high quality of the data that are produced by the low-cost optical technique is an important factor for the application of this technique in forest mapping. More studies are needed to explore the applicability for other types of forests. In general, the vertical fisheye technique is expected to be a feasible and efficient tool for documenting tree characteristics, mapping plots, and collecting tree data accurately. Augmenting the camera resolution is an easy alternative for improving point density and accuracy. This technique can be applied to many other mapping problems, such as indoor mapping and urban cadastral surveying.

## Figures and Tables

**Table 1:** Technical features of the fisheye camera.

Feature	Specification
Camera model	Nikon D3100
Nominal focal length	8 mm (Bower SLY 358N fisheye)
Pixel size	5.0 μm
Sensor dimensions	CMOS APS-C (23.1 mm × 15.4 mm)
Image dimensions	4608 pixels × 3072 pixels

**Table 2:** Technical features of the terrestrial laser scanning (TLS).

Feature	Specification
Model	Faro Photon3D X 330
Dimensions	240 × 100 × 200 mm
Weight	5.2 kg
Field of view	360° × 300°
Ranging error	2 mm (at 25 m distance)
Wavelength	1550 nm

**Table 3:** Differences between the measured DBH and the DBH estimated by circle fitting.

Trunk	Sigma (cm)	Error between Estimated and Measured DBHs (cm)
1	0.8	1.57
2	0.4	2.54
3	1.1	−1.13
4	0.4	−0.77
5	0.4	−1.94
6	0.4	0.22
7	0.5	0.76

**Table 4:** Point density of each trunk section and differences in the radii estimated by cylinder fitting.

Trunk	TLS-Based Point Cloud	Image-Based Point Cloud	Difference of Radius (cm)
	Number of Points	Number of Points	
1	7112	5427	0.31
2	15,703	6548	0.27
3	55,976	8415	0.48
4	9949	6432	0.94
5	18,924	7104	−0.66
6	1807	8739	1.51
7	28,532	6608	−0.22
Mean	19,714	7039	0.63
